# A Systematic Literature Review of Emerging Advances in Neurological Disorders: Diagnostic Innovations, Therapeutic Strategies, and Future Directions

**DOI:** 10.7759/cureus.112202

**Published:** 2026-07-07

**Authors:** V V Siva Ramireddy, Raja Gulfam Shaikh, Anisha R Pednekar, Dinesh Tripathi, Chhaya Trimbakrao Munde, Ketki Wagh

**Affiliations:** 1 Department of Neurosurgery, Santhiram Medical College, Nandyal, IND; 2 Department of Neurology, Super Speciality Hospital, Mahatma Gandhi Memorial Medical College, Indore, IND; 3 Department of Neurology and Neurosurgery, Goa Medical College and Hospital, Bambolim, IND; 4 Department of Biochemistry, Subharti Medical College, Meerut, IND; 5 Department of Physiology, Dr. D.Y. Patil College of Ayurved and Research Centre, Pimpri, IND; 6 Department of Preventive and Social Medicine, Dr. D.Y. Patil College of Ayurved and Research Centre, Pimpri, IND

**Keywords:** alzheimer’s disease, biomarkers, neurological disorders, precision neurology, therapeutic strategies

## Abstract

Neurological disorders remain a major clinical burden because they affect cognition, movement, vascular function, behavior, psychological health, and long-term independence. Recent advances in imaging, biomarkers, artificial intelligence, regenerative therapy, immunotherapy, and targeted pharmacology have expanded diagnostic and therapeutic possibilities, yet the evidence remains dispersed across different neurological conditions and study designs. This review aimed to synthesize emerging advances in neurological disorders, focusing on diagnostic innovations, therapeutic strategies, and future clinical directions. A systematic literature review approach was applied using the Preferred Reporting Items for Systematic Reviews and Meta-Analyses (PRISMA)-based screening principles. Eleven studies were included, covering ischemic stroke, glioblastoma, Alzheimer’s disease, multiple sclerosis, Parkinson’s disease, motor neuron disease, episodic migraine, transient ischemic attack, and postoperative delirium. Data were extracted on study design, condition, intervention or diagnostic method, comparator, outcomes, and key findings. Risk of bias was assessed using the Risk of Bias 2 (RoB 2) tool for randomized trials, the Risk of Bias in Non-randomized Studies of Interventions (ROBINS-I) for nonrandomized, uncontrolled, post hoc, feasibility, and biomarker-monitoring studies, and the Quality Assessment of Diagnostic Accuracy Studies-2 (QUADAS-2) for diagnostic and radiomics studies. Findings showed increasing use of radiomics, circulating tumor DNA, rhythm monitoring, vascular imaging, inflammatory markers, biologics, cell therapy, psychological intervention, and lipid-lowering therapy. Several studies reported promising clinical or biomarker signals, while others clarified treatment limitations in specific disease subtypes. Overall, the findings suggest an emerging shift toward precision-oriented neurology, but larger controlled trials, prospective biomarker validation, standardized outcomes, and longer follow-up are required before routine implementation.

## Introduction and background

Neurological disorders represent a major clinical and public health burden, affecting cognition, motor function, sensory function, vascular regulation, psychological health, functional independence, and quality of life [1,2]. This review does not treat all neurological diseases as one homogeneous entity. Instead, it applies a defined cross-condition framework focused on recent innovations in diagnostics, prognostics, biomarker- and imaging-based approaches, therapeutics, and supportive care relevant to precision neurology [3,4]. The included conditions, including ischemic stroke, transient ischemic attack (TIA), glioblastoma, Alzheimer’s disease (AD), Parkinson’s disease (PD), multiple sclerosis (MS), migraine, motor neuron disease (MND), and postoperative delirium (POD), were reviewed together because recent studies in these areas evaluated emerging approaches that may improve disease characterization, risk prediction, treatment selection, monitoring, and patient-relevant outcomes [1-5]. Although these disorders differ in etiology, pathophysiology, and clinical outcomes, their inclusion is justified by this shared translational framework. This framework was used to improve focus while retaining the review’s emphasis on shared diagnostic, prognostic, therapeutic, and translational themes across heterogeneous neurological conditions.

For clarity, precision neurology refers to the use of patient-specific clinical, imaging, molecular, biomarker, and functional data to guide diagnosis, prognosis, treatment selection, and monitoring. Radiomics refers to the extraction of quantitative features from medical images, while biomarkers are measurable biological indicators that may help identify disease activity, treatment response, or prognosis. The traditional diagnosis of neurological diseases has been based on clinical examination, symptom patterns, neuroimaging, electrophysiology, laboratory testing, and defined diagnostic criteria [4,6]. These strategies continue to be important, but in some cases, they are not effective enough to detect early disease, diagnose biological disease types, predict disease course, or inform treatment decisions for individuals [6,7]. Neurological diseases often share similar clinical characteristics, diverse disease courses, and differing responses to therapy, making clinical decision-making challenging [7,8]. This has increased interest in advanced diagnostic tools, including continuous physiological monitoring, circulating biomarkers, liquid biopsy, inflammatory markers, advanced MRI techniques, artificial intelligence (AI), and computer-aided diagnosis [8,9]. These tools may improve diagnostic accuracy and help detect structural, molecular, vascular, or functional changes before clinically evident deterioration occurs [9,10].

New opportunities for precision neurology have emerged recently based on advances in neuroimaging and computational analysis [9,10]. Radiomics can use routine imaging to generate quantitative features and translate these into a diagnostic or prognostic signature [9]. In some diseases, like AD and glioblastoma, slight anatomical changes or tumor-related changes can be important and carry valuable biological and clinical information [9,10]. Likewise, in stroke, vascular imaging and post-procedural imaging may detect complications, infarct patterns, and mechanisms of neurological worsening [2,10]. Consequently, cardiac rhythm monitoring after TIA or minor ischemic stroke may support secondary prevention by detecting AF, which can influence antithrombotic management [1,2]. The developments indicate an increasing shift in the future direction of neurological care toward a combination of clinical, imaging, molecular, and physiological data [6,2]. Despite their promise, these technologies remain limited by incomplete external validation, variable availability across clinical settings, high implementation cost, differences in data quality, and the need for standardized interpretation before routine use.

Therapeutic approaches for neurological diseases are also evolving [1-3]. Although the traditional approach has been to control symptoms, modify risk factors, or provide supportive care, the newer research efforts aim to identify disease mechanisms, including neuroinflammation, protein aggregation, immune dysregulation, vascular injury, mitochondrial dysfunction, excitotoxicity, blood-brain barrier disruption, and abnormal cellular repair [2,5]. Examples of recent study results are the neuroprotective strategies for POD, lipid-lowering and anti-inflammatory therapy in ischemic stroke, immunomodulation using stem cells in MS, monoclonal antibody for α-synuclein in PD, and molecularly targeted approaches in glioblastoma [1,6]. These methods have shifted from generic treatment to biologically informed and disease-subtype-specific interventions [6,8].

In addition, there is a growing focus on patient-centered and supportive-care approaches in the management of neurological diseases [7,8]. Numerous neurological disorders are chronic, progressive, or debilitating, and clinical benefit can be assessed only as a function of survival, imaging response, or laboratory change [3,7]. Meaningful neurological care focuses on outcomes like psychological adjustment, quality of life, functional independence, caregiver burden, fatigue, anxiety, depression, and treatment acceptability [7,8]. Psychological and behavioral interventions can thus be used in conjunction with biomedical therapy, especially when there are few therapeutic options for a disease [7].

Despite rapid progress in neurological research, the key knowledge gap is the lack of a unified synthesis showing how recent diagnostic, prognostic, biomarker-based, imaging-based, therapeutic, and supportive-care innovations are developing across major neurological disorders. Existing reviews usually focus on a single disease, technology, or treatment class, which limits understanding of shared precision-neurology trends, evidence strength, and remaining validation needs. This review addresses that gap by systematically summarizing selected recent studies through a common framework of diagnostic value, therapeutic mechanism, clinical outcomes, safety, risk of bias, and future research priorities.

Objective of the review

This systematic literature review examined recent diagnostic, prognostic, biomarker-based, imaging-based, therapeutic, and supportive-care advances in selected human neurological or neurologically relevant conditions, including ischemic stroke, TIA, glioblastoma, AD, PD, multiple sclerosis, migraine, MND, and POD. The interventions or index approaches included radiomics, vascular imaging, rhythm monitoring, circulating biomarkers, regenerative therapy, immunotherapy, targeted pharmacological therapy, and psychological intervention. Comparators included placebo, standard care, baseline status, disease-control groups, external comparator cohorts, or absence of the intervention, according to study design. Outcomes included diagnostic or prognostic performance, clinical response, functional outcome, survival, disease progression, symptom burden, safety, and patient-reported outcomes. Eligible studies included randomized controlled trials, phase I/II trials, post hoc analyses, diagnostic or prognostic modelling studies, biomarker studies, and feasibility studies published between 2021 and 2025. The review question was as follows: Among patients with selected neurological disorders, what recent diagnostic, prognostic, biomarker-based, imaging-based, therapeutic, and supportive-care innovations have been evaluated, and what evidence supports their clinical relevance, safety, and validation needs?

## Review

Methodology

Study Design

This review was designed as a systematic literature review with evidence mapping and narrative synthesis. The aim was not to estimate a pooled effect for one disease, intervention, or outcome, but to systematically identify, select, appraise, and synthesize recent studies evaluating emerging diagnostic, prognostic, biomarker-based, imaging-based, therapeutic, and supportive-care innovations across selected neurological disorders. This broader scope was justified by the review question, which focused on innovation type, translational relevance, and future validation needs rather than comparative effectiveness within a single neurological condition. The Preferred Reporting Items for Systematic Reviews and Meta-Analyses (PRISMA)-based screening and reporting principles were followed to maintain transparency in study identification, eligibility assessment, and evidence synthesis [11]. Eleven studies were included in the final synthesis, covering acute, chronic, neurodegenerative, neuroinflammatory, neuro-oncological, vascular, headache-related, motor neuron, and perioperative neurocognitive conditions.

Search Strategy

A systematic literature search was conducted to find relevant studies on advances in diagnosis, prognosis, and treatment in neurological diseases. The databases searched included PubMed/MEDLINE, Embase, Scopus, Web of Science, and Google Scholar. The overall search period covered articles published from 2021 to 2025. The literature search was conducted for recent clinical, translational, diagnostic, interventional, and biomarker-based studies. Search terms were created in three main areas: neurological disorder, diagnostic innovation, and therapeutic advancement. The search strategy combined controlled vocabulary terms and free-text keywords related to neurological disorders, biomarkers, radiomics, AI, neuroimaging, precision neurology, therapeutic strategies, immunotherapy, regenerative therapy, and clinical outcomes. Boolean operators were used to combine terms within and across these domains. In addition, reference lists of eligible articles were checked as a source for finding more relevant articles. Only research papers that were directly related to the title of the review and the focus of the research were taken into consideration for final inclusion. Because the review question focused on emerging innovations rather than one intervention or disease subtype, the search strategy intentionally captured multiple evidence streams, including diagnostic, prognostic, biomarker-based, imaging-based, interventional, and supportive-care studies. The electronic database search strategies used to identify eligible studies are summarized in Table 1.

**Table 1:** Electronic database search strategies.

Database	Condensed search strategy
PubMed/MEDLINE	("neurological disorders" OR stroke OR TIA OR Alzheimer* OR Parkinson* OR "multiple sclerosis" OR glioblastoma OR migraine OR "motor neuron disease") AND (diagnos* OR prognos* OR biomarker* OR radiomic* OR neuroimaging OR "artificial intelligence") AND (treatment OR intervention OR immunotherapy OR "stem cell*" OR "monoclonal antibod*" OR "PARP inhibitor*" OR "PCSK9 inhibitor*" OR insulin OR onabotulinumtoxinA)
Embase	('neurological disease'/exp OR stroke OR TIA OR Alzheimer* OR Parkinson* OR 'multiple sclerosis' OR glioblastoma OR migraine OR 'motor neuron disease') AND ('diagnosis'/exp OR prognos* OR biomarker* OR radiomic* OR neuroimaging OR 'artificial intelligence') AND ('therapy'/exp OR treatment OR intervention OR immunotherapy OR 'stem cell*' OR 'monoclonal antibody' OR 'PARP inhibitor*' OR 'PCSK9 inhibitor*' OR insulin OR onabotulinumtoxinA)
Scopus	TITLE-ABS-KEY("neurological disorder" OR stroke OR TIA OR Alzheimer* OR Parkinson* OR "multiple sclerosis" OR glioblastoma OR migraine OR "motor neuron disease") AND TITLE-ABS-KEY(diagnos* OR prognos* OR biomarker* OR radiomic* OR neuroimaging OR "artificial intelligence") AND TITLE-ABS-KEY(treatment OR intervention OR immunotherapy OR "stem cell*" OR "monoclonal antibod*" OR "PARP inhibitor*" OR "PCSK9 inhibitor*" OR insulin OR onabotulinumtoxinA)
Web of Science	TS=("neurological disorder" OR stroke OR TIA OR Alzheimer* OR Parkinson* OR "multiple sclerosis" OR glioblastoma OR migraine OR "motor neuron disease") AND TS=(diagnos* OR prognos* OR biomarker* OR radiomic* OR neuroimaging OR "artificial intelligence") AND TS=(treatment OR intervention OR immunotherapy OR "stem cell*" OR "monoclonal antibod*" OR "PARP inhibitor*" OR "PCSK9 inhibitor*" OR insulin OR onabotulinumtoxinA)
Google Scholar	("neurological disorder" OR stroke OR TIA OR Alzheimer* OR Parkinson* OR "multiple sclerosis" OR glioblastoma OR migraine OR "motor neuron disease") AND (diagnos* OR prognos* OR biomarker* OR radiomic* OR neuroimaging OR "artificial intelligence") AND (treatment OR intervention OR immunotherapy OR "stem cell*" OR "monoclonal antibody*" OR "PARP inhibitor*" OR "PCSK9 inhibitor*" OR insulin OR onabotulinumtoxinA)

Eligibility Criteria

Studies were included if they examined human participants with selected neurological disorders or neurologically relevant perioperative outcomes and evaluated emerging diagnostic, prognostic, biomarker-based, imaging-based, AI/radiomics-based, therapeutic, preventive, regenerative, pharmacological, immunomodulatory, or supportive-care strategies. Eligible conditions included acute cerebrovascular, neuro-oncological, neurodegenerative, neuroinflammatory, demyelinating, movement, headache-related, motor neuron, and perioperative neurocognitive disorders.

Eligible study designs included randomized controlled trials, phase I/II trials, post hoc analyses, open-label studies, feasibility studies, diagnostic or prognostic modeling studies, radiomics studies, biomarker-based studies, cohort studies, and multi-cohort diagnostic studies. These designs were included to capture different stages of clinical translation, from comparative efficacy and safety to feasibility, early therapeutic signals, diagnostic classification, and prognostic performance. Evidence was not pooled across heterogeneous designs; conclusions were synthesized narratively and interpreted according to study design, comparator type, outcome domain, sample size, and risk of bias. Studies were eligible only if they reported clinically relevant diagnostic, prognostic, therapeutic, functional, imaging, biomarker, safety, or patient-reported outcomes, were published between 2021 and 2025, and were available as full-text articles. Exclusion criteria included non-neurological focus, absence of diagnostic/prognostic or therapeutic innovation, insufficient outcome data, publication outside the search period, conference abstracts, editorials, letters, narrative reviews, protocols without results, animal-only or in vitro studies, non-English articles, and duplicate records.

Study Selection

All identified records were screened for relevance using a stepwise process. Two reviewers independently screened the titles and abstracts of all retrieved records against the predefined eligibility criteria. Studies considered potentially relevant by either reviewer were advanced to full-text assessment. The same two reviewers then independently reviewed the full-text articles to determine final eligibility. Any disagreements during title/abstract screening or full-text assessment were resolved through discussion, and unresolved disagreements were adjudicated by a third reviewer. Studies that met the established inclusion criteria and demonstrated relevance to diagnostic innovation, therapeutic strategy, or future directions in neurology were included. The final included studies were organized according to neurological condition, study design, intervention or diagnostic/prognostic approach, comparator, outcomes evaluated, and main findings. A PRISMA flow diagram was used to summarize the number of records identified, records remaining after duplicate removal, titles and abstracts screened, full-text articles assessed for eligibility, full-text articles excluded with reasons, and studies included in the final synthesis.

Data Extraction

A structured format for systematic evidence synthesis was used to extract data. Data extraction was performed independently by two reviewers using a predefined data extraction form. Extracted information included author name, year of publication, neurological condition, study design, sample characteristics, intervention or diagnostic/prognostic approach, comparator/control group, main outcomes, key findings, and relevance to the objective of the review. Extracted data were cross-checked between reviewers to ensure consistency and completeness. Any discrepancies were resolved by discussion, with consultation from a third reviewer when necessary. The primary goal of data extraction was to determine the key clinical or translational contribution of each study. The results were subsequently tabulated into a main characteristics table, which allowed for a clear comparison of the included studies. The strength of conclusions was interpreted qualitatively according to evidence level, with stronger inference given to randomized controlled trials and blinded comparative studies, and more cautious interpretation applied to early-phase, uncontrolled, feasibility-based, post hoc, diagnostic-modeling, and biomarker-based studies.

Outcomes of Interest

The principal outcome measures were measures reported in the included studies that were of interest for diagnosis, prognosis, therapy, function, and/or safety. Diagnostic/prognostic endpoints comprised the imaging-based classification, the radiomics performance, the biomarker response, the detection of atrial fibrillation (AF), the detection of infarcts, and the prediction of clinical progression. Therapeutic outcomes included changes in neurological deterioration and the presence of delirium, progression of motor status, frequency of migraine, survival, progression-free survival, quality of life, psychological outcomes, inflammatory markers, and adverse events. The results were interpreted based on the original objectives and design of the studies included.

Risk-of-Bias Assessment

Risk of bias was assessed using study design-specific tools. Randomized controlled trials were assessed using the Cochrane Risk of Bias 2 (RoB 2) tool [12]. Nonrandomized intervention studies, open-label clinical studies, post hoc analyses of intervention trials, biomarker-monitoring studies, and uncontrolled feasibility studies were assessed using the Risk of Bias in Non-randomized Studies of Interventions (ROBINS-I) [13]. Diagnostic accuracy, imaging-based classification, and radiomics studies were assessed using the Quality Assessment of Diagnostic Accuracy Studies-2 (QUADAS-2) [14]. The Prediction Model Risk of Bias Assessment Tool (PROBAST) was not applied because none of the included studies primarily developed or validated a formal prediction model. Selection bias, confounding, measurement bias, outcome assessment, reporting bias, and overall risk were evaluated according to the domains of the relevant tool. Each study was assessed with the tool most appropriate to its design. Biomarker-monitoring and prognostic studies without formal prediction-model development were assessed using ROBINS-I rather than PROBAST. Randomized and blinded trials were generally considered to have a lower risk of bias, while uncontrolled, small-sample, open-label, post hoc, diagnostic, radiomics, and biomarker-based studies were interpreted cautiously according to their design-specific limitations.

Data Synthesis

A structured narrative synthesis with evidence mapping was performed because the included studies differed substantially in neurological condition, design, intervention or index approach, comparator, outcome definition, and follow-up duration. Quantitative meta-analysis was not performed because clinical and methodological heterogeneity made pooled effect estimation inappropriate. The synthesis was organized around the predefined review framework of diagnostic and prognostic innovations, therapeutic strategies, and future clinical directions. Study findings were compared according to condition, innovation type, study design, outcome domain, safety findings, and risk-of-bias assessment. Numerical summaries and figures were used only to illustrate the distribution of evidence themes and research priorities, not to imply statistical pooling or comparative effectiveness.

Results

Study Selection

**Figure 1:**
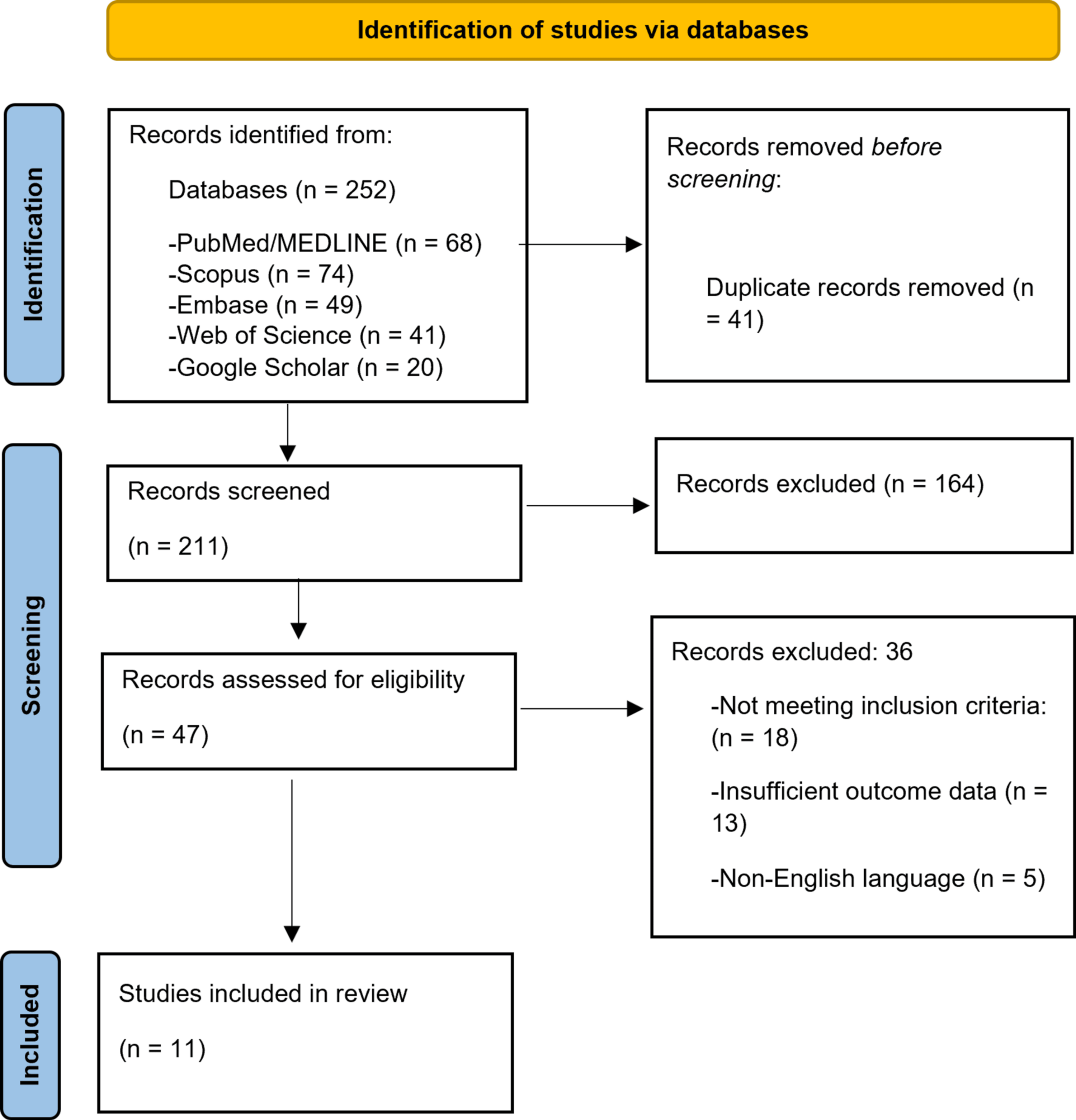
PRISMA flow diagram. PRISMA: Preferred Reporting Items for Systematic Reviews and Meta-Analyses.

Eleven studies were found through the search and screening of the databases. In accordance with the PRISMA flow diagram, a total of 252 records were identified from electronic databases, including PubMed/MEDLINE (n = 68), Scopus (n = 74), Embase (n = 49), Web of Science (n = 41), and Google Scholar (n = 20). After the removal of 41 duplicate records, 211 records were screened by title and abstract. Of these, 164 records were excluded because they were not relevant to the review objective, did not focus on neurological disorders, or did not evaluate diagnostic, prognostic, or therapeutic advances. A total of 47 records were assessed for eligibility through full-text review, and 36 were excluded because they did not meet the inclusion criteria (n = 18), had insufficient outcome data (n = 13), or were non-English publications (n = 5). The final corpus included studies on POD, ischemic stroke, glioblastoma, AD, secondary progressive MS, TIA, PD, MND, episodic migraine, and acute non-cardiogenic ischemic stroke. A total of 11 studies were included in the final synthesis. Evidence consisted of randomized controlled trials, phase 1 or 2 studies, post hoc analyses, uncontrolled feasibility studies, and diagnostic radiomics and biomarker-based studies. The process of selecting, excluding, and finalizing the 11 studies is summarized as a PRISMA flow diagram (Figure 1).

Study Characteristics

The 11 studies reviewed included innovations in diagnosis, treatment, and future directions in a variety of neurological diseases. These interventions included intranasal insulin, placenta-derived mesenchymal stem cells, prasinezumab, acceptance and commitment therapy, onabotulinumtoxinA, evolocumab, atorvastatin, and pamiparib-based regimens, along with biomarker and immune-related monitoring approaches. Diagnostic and prognostic approaches included imaging-defined infarcts after thrombectomy, AF detection, hippocampal radiomics, and tumor in situ fluid circulating tumor DNA (ctDNA) monitoring. Delirium, neurological deterioration, survival, progression-free survival, motor progression, cognition, quality of life, functional recovery, inflammatory markers, and safety were outcomes. The features of the study designs, study populations, interventions, comparators, outcomes, and clinically salient results are described in Table 2.

**Table 2:** Summary of included studies. ACT: acceptance and commitment therapy; AD: Alzheimer’s disease; AE: adverse event; AF: atrial fibrillation/flutter; AIS: acute ischemic stroke; ALS: amyotrophic lateral sclerosis; AUC: area under the curve; CM: chronic migraine; ctDNA: circulating tumor DNA; DTI: diffusion tensor imaging; EDSS: Expanded Disability Status Scale; EM: episodic migraine; END: early neurological deterioration; fMRI: functional magnetic resonance imaging; IL: interleukin; INT: infarct in a new territory; LAA: large artery atherosclerosis; LDL-C: low-density lipoprotein cholesterol; MDS-UPDRS: Movement Disorder Society-sponsored revision of the Unified Parkinson’s Disease Rating Scale; MND: motor neuron disease; MRI: magnetic resonance imaging; mRS: modified Rankin Scale; MS: multiple sclerosis; NIHSS: National Institutes of Health Stroke Scale; onabotA: onabotulinumtoxinA; ORR: objective response rate; OS: overall survival; PD: Parkinson’s disease; PFS: progression-free survival; PLMSC: placenta-derived mesenchymal stem cell; POD: postoperative delirium; PPMI: Parkinson’s Progression Markers Initiative; PROs: patient-reported outcomes; QoL: quality of life; RCT: randomized controlled trial; SLR: systematic literature review; SVO: small vessel occlusion; TIA: transient ischemic attack; TMB: tumor mutational burden; TNF-α: tumor necrosis factor-alpha.

Study	Neurological condition	Study design/sample	Diagnostic innovation/therapeutic strategy	Comparator/control	Main outcomes	Key findings	Relevance
Wang et al. [15]	Postoperative delirium in elderly hip-fracture patients	Randomized, placebo-controlled, double-blind trial; 96 enrolled, 92 completed	Perioperative intranasal insulin, 20 U twice daily from 2 days preoperatively to 2 days postoperatively	Intranasal saline placebo	POD within 5 postoperative days; IL-6 and CRP	Intranasal insulin reduced POD incidence versus placebo: 10.9% vs. 30.4%, P=0.020. IL-6 and CRP were also lower on postoperative day 1; no intervention-related adverse events were reported.	Emerging preventive neuroprotective strategy targeting postoperative neuroinflammation
Singh et al. [16]	Acute ischemic stroke treated with endovascular thrombectomy	Post hoc analysis from ESCAPE-NA1; n = 1,092	Imaging-based identification of infarcts in a new territory after thrombectomy	INT vs. no INT	INT incidence; 90-day mRS; mortality	INT occurred in 103 patients, about 9.3-9.4%. INT was associated with poorer 90-day functional independence, adjusted RR = 0.71, and higher death risk, adjusted RR = 2.15.	Diagnostic/prognostic imaging marker for procedural complications in stroke thrombectomy
Piotrowski et al. [17]	Treatment-naïve or recurrent/refractory glioblastoma	Phase Ib/II open-label dose-escalation/expansion trial; n = 116	Pamiparib, a PARP1/2 inhibitor, combined with radiotherapy and/or low-dose temozolomide	No randomized control; compared with protocol/historical benchmarks	Safety, dose-limiting toxicity, disease control, ORR, PFS, OS	Combination regimens were tolerable. In newly diagnosed glioblastoma, disease control was 67.9%, ORR was 11.3%, and median OS was 12.8 months; in recurrent/refractory disease, disease control was 40.9%, ORR was 13.6%, and median OS was 7.3 months.	Novel DNA-repair-targeted therapeutic strategy for glioblastoma
Guo et al. [18]	Recurrent glioblastoma	Open-label phase II trial; n = 32	Tumor in situ fluid ctDNA monitoring during tislelizumab plus low-dose bevacizumab	No control arm	PFS, OS, ORR, ctDNA/TMB dynamics	Median PFS was 8.2 months, median OS was 14.3 months, 12-month OS was 43.8%, and ORR was 56.3%. A >20% decrease in mutant allele fraction and TMB after treatment was associated with better prognosis.	Precision oncology biomarker strategy for monitoring immunotherapy response
Xia et al. [19]	Alzheimer’s disease	Retrospective multi-cohort radiomics and radiogenomics study; discovery, n = 420; radiogenomics, n = 266	MRI-based hippocampal radiomics model plus peripheral blood transcriptomic mapping	AD vs. normal controls across validation cohorts	AD classification; AUC; biological pathway correlation	A 12-feature hippocampal radiomics model accurately identified AD, with AUCs of 0.929, 0.907, and 0.874 in validation cohorts. Radiogenomics linked the model to myeloid leukocyte and neutrophil activation pathways.	Diagnostic innovation combining neuroimaging, AI/radiomics, and biological interpretation
Shokati et al. [20]	Secondary progressive multiple sclerosis	Open-label phase I clinical trial; n = 5	Intravenous placenta-derived mesenchymal stem cells, 3×10⁶ cells/kg	No control arm	Safety, EDSS, cognition, DTI/fMRI, cytokines, CD20/CD19 markers	PLMSC transplantation was feasible and generally safe; only transient headache was reported in two patients. Exploratory outcomes suggested EDSS improvement, reduced inflammatory cytokines, increased IL-10, reduced CD20/CD19 B-cell markers, and improved fMRI connectivity.	Regenerative and immunomodulatory cell therapy for progressive MS
Kamel et al. [21]	TIA and minor ischemic stroke	Secondary analysis of the POINT trial; n = 4,832 after excluding baseline AF	Detection of newly diagnosed atrial fibrillation/flutter after TIA vs. minor stroke	TIA vs. minor ischemic stroke	New AF diagnosis within 90 days; recurrent ischemic stroke	AF risk was similar after stroke and TIA using the original definition: 2.7% vs. 2.0%, P=0.15. After imaging-based reclassification, AF risk was higher after stroke: 2.7% vs. 1.8%, P=0.04, but event type had poor predictive utility.	Diagnostic strategy supporting similar rhythm-monitoring approaches after TIA and minor stroke
Pagano et al. [22]	Early Parkinson’s disease	Exploratory open-label extension analysis of PASADENA; PASADENA, n = 271; external PPMI comparator, n = 303	Prasinezumab, anti-α-synuclein monoclonal antibody	External comparator from the PPMI observational cohort	4-year MDS-UPDRS Part III OFF/ON and Part II progression	Prasinezumab-treated delayed- and early-start groups showed slower motor progression than the external comparator, including lower MDS-UPDRS Part III OFF, ON, and Part II progression over 4 years. Findings were exploratory and require confirmation.	Disease-modifying biologic strategy targeting α-synuclein spread in Parkinson’s disease
Gould et al. [23]	Motor neuron disease/ALS	Uncontrolled feasibility study; n = 29 people with MND	Acceptance and Commitment Therapy, up to 8 one-to-one sessions plus usual care	No control arm	Recruitment, engagement, acceptability, QoL, anxiety, depression	Recruitment and engagement targets were met: 29/28 targets recruited and 76% completed at least two sessions. ACT was acceptable, with possible small improvements in anxiety and psychological QoL despite functional decline.	Psychotherapeutic and supportive-care innovation for neurodegenerative disease
Pozo-Rosich et al. [24]	Episodic migraine	Phase 3 multicenter randomized, double-blind, placebo-controlled PRECLUDE trial; n = 775	OnabotulinumtoxinA 155 U or 195 U for migraine prevention	Placebo	Monthly migraine days, headache days, responder rate, acute medication days, PROs, safety	OnabotA was well tolerated but did not significantly reduce monthly migraine days versus placebo. Mean change in monthly migraine days was similar: placebo −3.6, onabotA 155 U −3.5, onabotA 195 U −3.4.	Negative phase 3 evidence refining therapeutic boundaries between episodic and chronic migraine
Liu et al. [25]	Acute non-cardiogenic ischemic stroke with or without large artery atherosclerosis	Post hoc subgroup analysis of randomized trial; n = 272	Evolocumab plus atorvastatin for prevention of early neurological deterioration	Atorvastatin monotherapy	END within 7 days; LDL-C target achievement; IL-6; 90-day mRS	Evolocumab plus atorvastatin reduced END in LAA stroke: 14.0% vs. 28.0%, RR = 0.45, P = 0.006, but not in SVO stroke. Favorable 90-day mRS was also higher in LAA patients: 81.7% vs. 61.3%, P < 0.001.	Acute stroke subtype-specific lipid-lowering and anti-inflammatory therapeutic strategy

Cross-study synthesis showed different levels of clinical maturity across the included studies. Randomized placebo-controlled trials on intranasal insulin for POD and onabotulinumtoxinA for episodic migraine provided the strongest clinical evidence. Stroke studies complemented each other by linking imaging-defined infarcts, rhythm monitoring, lipid-lowering therapy, inflammatory response, and functional outcomes within a precision vascular-neurology framework. Neuro-oncology studies combined targeted therapy with biomarker monitoring, while neurodegenerative and neuroinflammatory studies mainly provided exploratory evidence on α-synuclein targeting, placenta-derived mesenchymal stem cells, and psychological support. Across categories, the main evidence gaps were small sample size, limited external validation, variable follow-up, heterogeneous outcomes, and limited multicenter replication. Overall, Table 2 compares innovation type, evidence maturity, outcome relevance, and validation needs across neurological conditions.

Risk-of-Bias Assessment

Risk of bias was assessed using tools selected according to study design. Randomized controlled trials were assessed with RoB 2, nonrandomized and uncontrolled intervention studies with ROBINS-I, and diagnostic and radiomics studies with QUADAS-2. PROBAST was not applied because none of the included studies primarily developed or validated a formal prediction model. Biomarker-monitoring, prognostic, post hoc, open-label, and uncontrolled studies were assessed using ROBINS-I according to their primary design. The assessment considered key sources of bias, including selection bias, confounding, measurement bias, outcome assessment, and reporting bias. Randomized and blinded studies were generally judged to have a lower risk of bias, while uncontrolled, small-sample, open-label, post hoc, diagnostic, radiomics, and biomarker-based studies were interpreted with caution because of their design-specific limitations. The overall judgments are summarized in Table 3.

**Table 3:** Risk-of-bias assessment of included studies. RoB 2: Risk of Bias 2; ROBINS-I: Risk of Bias in Non-randomized Studies of Interventions; QUADAS-2: Quality Assessment of Diagnostic Accuracy Studies-2.

Study	Tool used	Rationale for tool selection	Selection bias	Measurement bias	Reporting bias	Overall risk
Wang et al. [15]	RoB 2	Randomized, placebo-controlled, double-blind trial	Low	Low	Low	Low
Singh et al. [16]	ROBINS-I	Post hoc nonrandomized prognostic analysis of trial data	Moderate	Moderate	Low	Moderate
Piotrowski et al. [17]	ROBINS-I	Open-label phase Ib/II intervention study without a randomized control	Moderate	Moderate	Low	Moderate
Guo et al. [18]	ROBINS-I	Open-label biomarker-monitoring/prognostic study without formal prediction-model development	Moderate	Moderate	Low	Moderate
Xia et al. [19]	QUADAS-2	Radiomics-based diagnostic classification study	Moderate	Moderate	Low	Moderate
Shokati et al. [20]	ROBINS-I	Open-label phase I uncontrolled intervention study	High	Moderate	Low	High
Kamel et al. [21]	ROBINS-I	Secondary nonrandomized analysis of trial data	Moderate	Moderate	Low	Moderate
Pagano et al. [22]	ROBINS-I	Open-label extension with external comparator cohort	Moderate	Moderate	Low	Moderate
Gould et al. [23]	ROBINS-I	Uncontrolled feasibility study	High	Moderate	Low	High
Pozo-Rosich et al. [24]	RoB 2	Randomized, placebo-controlled, double-blind trial	Low	Low	Low	Low
Liu et al. [25]	ROBINS-I	Post hoc subgroup analysis of randomized trial data	Moderate	Moderate	Low	Moderate

Diagnostic and Prognostic Innovations

**Figure 2:**
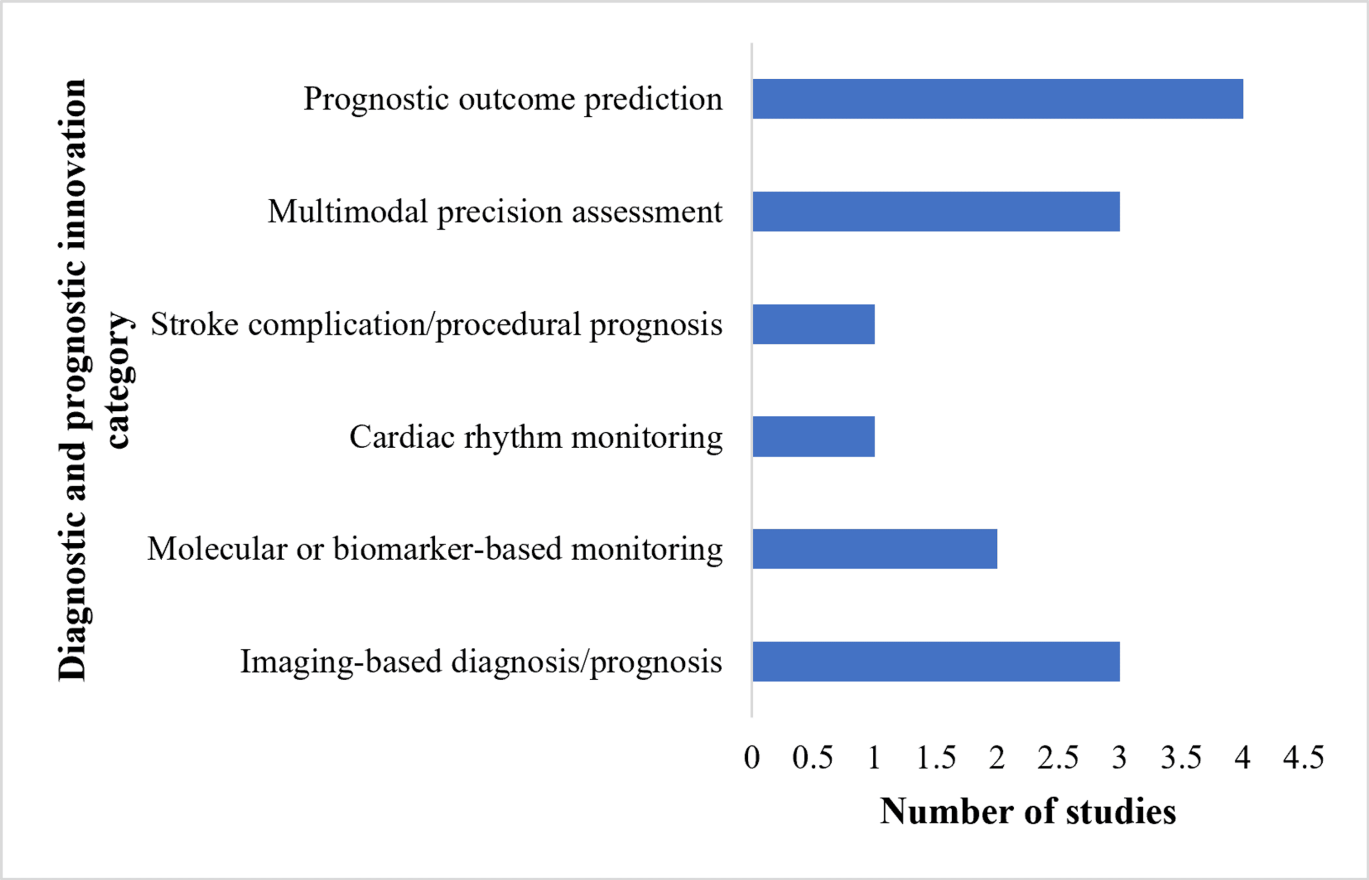
Diagnostic and prognostic innovation categories.

The included studies suggested exploratory diagnostic and prognostic trends relevant to precision neurology, but the evidence should be interpreted cautiously because the review included only 11 studies across heterogeneous neurological conditions. In AD, hippocampal radiomics showed potential for linking MRI-derived features with biological pathway signatures. In glioblastoma, tumor in situ fluid ctDNA was evaluated as a biomarker-monitoring approach during bevacizumab-based therapy and immunotherapy. Stroke-related studies assessed imaging-defined infarcts after thrombectomy and AF detection after TIA or minor ischemic stroke. These findings suggest that imaging, molecular markers, vascular monitoring, and longitudinal risk assessment may contribute to future neurological classification and prognosis, but they do not establish broad clinical applicability across neurology as a whole. Larger condition-specific studies, external validation, and standardized outcome measures are required before these approaches can be recommended for routine clinical use. Figure 2 summarizes the diagnostic and prognostic innovation categories represented in the included studies.

Therapeutic Strategies and Future Directions

**Figure 3:**
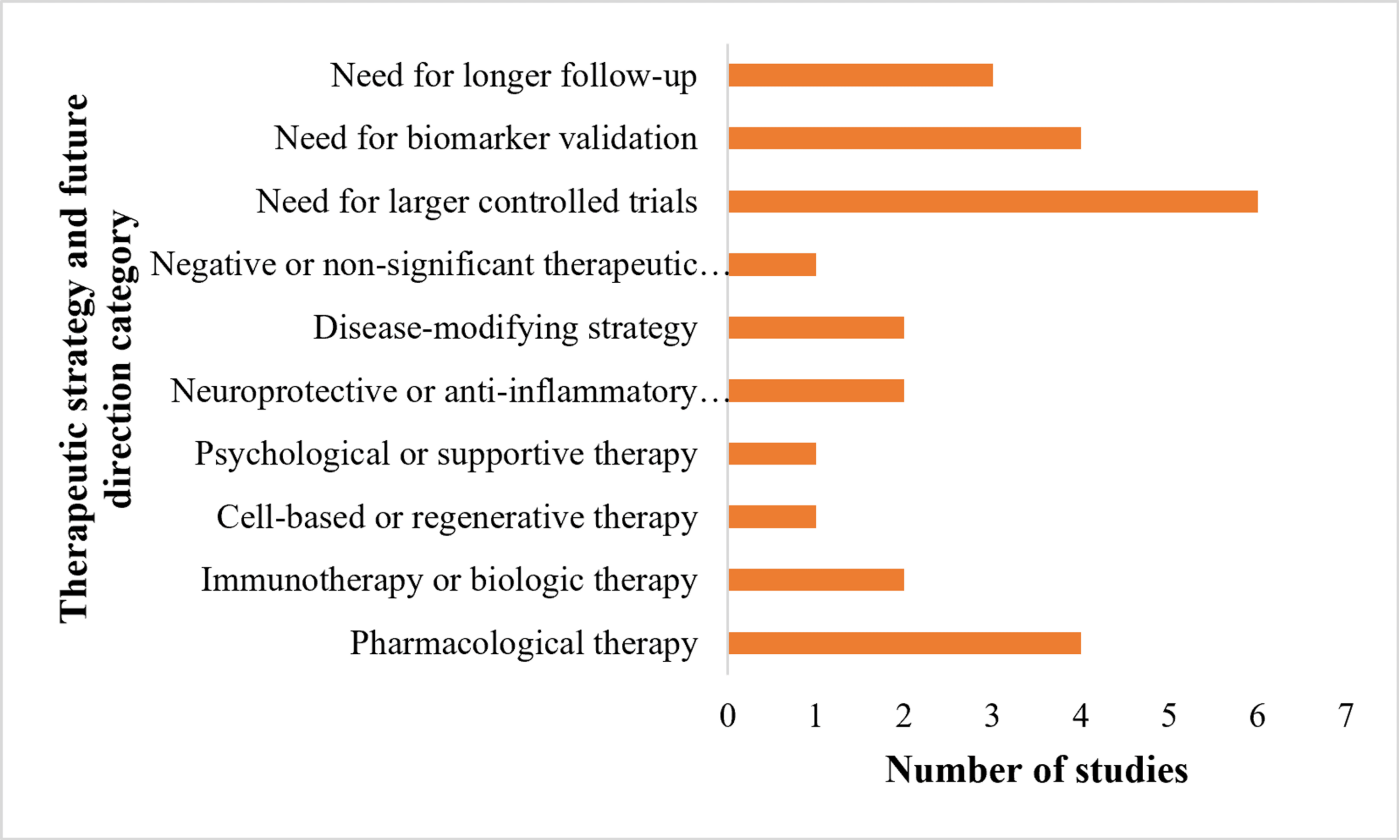
Therapeutic strategies and future directions.

The included therapeutic studies suggested increasing interest in mechanism-based interventions. Intranasal insulin was evaluated for perioperative neuroinflammation and delirium prevention, while evolocumab plus atorvastatin was assessed for lipid-related and inflammatory pathways linked to early neurological deterioration in large artery atherosclerosis stroke. The immunomodulation of placenta-derived mesenchymal stem cells in secondary progressive MS and prasinezumab on the propagation of α-synuclein in PD was explored. Acceptance and commitment therapy was explored as a psychological supportive-care strategy for people with MND. OnabotulinumtoxinA was shown to be safe and not effective in episodic migraine, which suggests treatment boundaries between migraine subtypes. Larger controlled trials, validation of biomarkers, longer follow-up, and incorporation of patient-centered outcomes into precision neurological care across the spectrum of neurological disease and settings should be prioritized for future research. Figure 3 summarizes the main future research priorities identified across the included studies, with larger controlled trials being the most frequently represented need.

Discussion

This systematic literature review provides a focused synthesis of selected recent diagnostic and therapeutic innovations across heterogeneous neurological conditions. The use of an integrated approach (imaging, molecular markers, targeted therapeutics, immune modulation, patient-centered outcomes) over single-domain clinical assessment has been a growing trend in the presented evidence, reflecting the evolution of neurological science. This is in line with the general trend and evolution of advanced neurological interventions and precision-based treatment models reported in the recent literature [26,27].

The diagnostic and prognostic results suggest that multimodal disease characterization may gain increasing relevance in neurological assessment in the future. Imaging-based studies showed that structural or procedural imaging data can be converted to clinically meaningful imaging risk markers, such as radiomic analysis or vascular imaging analysis. In addition to being applied to the development of a noninvasive diagnostic model for AD, hippocampal radiomics were also applied to link imaging signatures to biological pathways. The studies on stroke showed that post-thrombectomy infarcts in new territories and the presence of new AF following TIA or minor stroke required post-stroke surveillance and risk stratification based on the stroke mechanism. These outcomes are consistent with the potential role of pattern recognition, predictive analytics, and AI-supported diagnostic approaches in neurology and emergency care.

A second major trend was the application of biomarkers as a tool for prediction and as a therapeutic monitoring tool. The glioblastoma studies suggested the potential of molecular surveillance, including tumor in situ fluid ctDNA, for monitoring treatment response and prognosis. This is indicative of a trend in neuro-oncology from a purely radiological follow-up to molecularly informed monitoring. Similar biomarker thinking was seen in the inflammatory and vascular studies in which the treatment effect was evaluated based on cytokines, lipid markers, and immune-related outcomes. These methods emphasize the importance of using clinical outcomes in conjunction with biological assessments for the interpretation of response to treatment.

The therapeutic studies suggested preliminary evidence for mechanism-based interventions. Of the four drug candidates that were highlighted, intranasal insulin was used for its role in POD and for showing promise for the treatment of acute ischemic stroke, as well as for PD (evolocumab plus atorvastatin) and secondary progressive MS (placenta-derived mesenchymal stem cells). These mechanisms included neuroinflammation, vascular system damage, lipid-related damage to the vascular system, propagation of α-synuclein, dysfunction of the immune system, and regenerative repair. This is similar to the current methods of treating neurological diseases, where identification of disease pathways is key to therapeutic development, and targeted intervention is the key approach.

The supplement also includes studies that show the impact of disease type and biological context on treatment response. The evolocumab subgroup analysis indicated that the effect of evolocumab did not appear to be the same across stroke subtypes, with patients with large artery atherosclerosis showing greater improvements than those with small vessel occlusion. Similarly, the PRECLUDE trial showed that onabotulinumtoxinA, which is effective in chronic migraine, was ineffective in episodic migraine [24]. This distinction supports the concept that clinical diagnosis may be inadequate and therapeutic response may be associated with neurobiologic phenotype, stage, and/or suitability for a particular mechanism.

The neurodegenerative and neuroinflammatory studies also highlight the shift to disease-modifying interventions. During long-term follow-up, plasma-derived prasinezumab was associated with a decreased motor progression rate in PD, and PMSCs in MS showed exploratory signals of the immune and imaging pathways. The findings are consistent with other developments in the field of neurological therapeutics, including the development of biologics, cell-based therapy, nanotechnology-based delivery, and new molecular platforms [28,29]. The evidence was varied across all these studies, but in general, they show the direction of innovation during this time in the treatment of chronic neurological diseases.

The evidence also included patient-centered care. As part of MND, acceptance and commitment therapy (ACT) targets psychological flexibility, anxiety, and quality of life. Therapeutic advances in the field of neurology should not be solely drug-based. Neurological disorders can result in long-term disabilities affecting functioning, emotions, and caregiver stress. Thus, psychological outcomes are part of a clinically measurable model of neurological care, requiring additional support measures to be a part of it. This reflects the new paradigm that has emerged, focusing on the integration of biomarkers (along with imaging, neuroinflammation, and functional outcome) in the evaluation of neurodegenerative disease [30].

Overall, the included studies suggest an emerging movement toward precision-oriented neurological care, but the evidence remains preliminary and heterogeneous. The strongest translational signal was observed in studies that integrated diagnostic tools, biological mechanisms, and therapeutic or monitoring approaches. Disease activity, the response to treatment, and clinical benefit were repeatedly defined using imaging, molecular biomarkers, immune pathways, and functional outcomes. This multifaceted approach may support future development of more personalized diagnostic and therapeutic models based on disease mechanism, subtype, and patient-specific clinical need [27-31].

Limitations and Future Directions

There are some limitations of this review. Because of the broad clinical heterogeneity across neurological conditions, study designs, intervention types, diagnostic methods, outcome definitions, and follow-up durations, quantitative meta-analysis was not performed. The review protocol was not prospectively registered in the International Prospective Register of Systematic Reviews (PROSPERO) or an equivalent registry, which limits external verification of the prespecified methodology and may increase the risk of selection or reporting bias. The evidence base was also small, with only 11 included studies across multiple neurological conditions, so the findings should be interpreted as exploratory rather than definitive. Although the included studies were recent and relevant, they may not capture the full breadth of neurological advances from 2021 to 2025, particularly in rapidly evolving areas such as AD, PD, MS, and AI-based neurology. Several included studies were early-phase, open-label, uncontrolled, feasibility-based, or post hoc analyses, which limited causal interpretation. Some studies had small sample sizes, external comparator groups, or exploratory biomarker endpoints, which may increase selection bias, confounding, and limited generalizability. The restriction to English-language full-text publications may also have introduced language bias, and publication bias could not be excluded because of the small and heterogeneous evidence base.

Large, multicenter, randomized controlled trials are needed to establish therapeutic efficacy, safety, and clinical applicability. Prospective validation of diagnostic and prognostic tools, including radiomics, ctDNA, inflammatory biomarkers, and rhythm-monitoring strategies, is needed before routine clinical use. Standardized outcomes, longer follow-up, disease-subtype stratification, and patient-centered measures are needed to support the translation of precision neurology into clinical practice. Future reviews should include larger condition-specific evidence bases, protocol registration, broader language inclusion where feasible, and formal assessment of publication bias when sufficient studies are available.

## Conclusions

This systematic literature review highlights the growing focus on precision-based diagnosis, mechanism-based therapy, and patient-centered care in recent advances for neurological disorders. The included studies suggested potential roles for diagnostic and prognostic approaches such as radiomics, molecular monitoring, vascular imaging, atrial fibrillation detection, and biomarker-based outcome assessment. The evaluated therapies included neuroprotective, immunomodulatory, regenerative, psychological, lipid-lowering, and targeted oncological strategies. Overall, the findings indicate an emerging movement toward integrated neurological care using imaging, molecular information, disease phenotype, and functional outcomes, but the evidence remains preliminary. Most findings should be interpreted as promising signals rather than established clinical effects because several studies were early-phase, open-label, feasibility-based, post hoc, biomarker-focused, or conducted in small cohorts. Promising findings were observed for POD prevention, early neurological deterioration after ischemic stroke, motor progression monitoring in PD, immunomodulation in MS, biomarker monitoring in glioblastoma, and psychological support in MND. The negative episodic migraine trial also helped clarify the therapeutic boundaries of onabotulinumtoxinA in episodic migraine. These findings support further investigation of precision-neurology approaches, but larger controlled studies, standardized outcomes, longer follow-up, and external validation are required before firm clinical conclusions or widespread implementation can be recommended.

## Appendices

The PICO (population, intervention, comparison, outcome) framework used to guide study selection and evidence synthesis is presented in Table 4, detailing the population, intervention or index approach, comparator, and outcomes for each included study.

**Table 4:** PICO framework of included studies. AF: atrial fibrillation; DTI: diffusion tensor imaging; EDSS: Expanded Disability Status Scale; fMRI: functional magnetic resonance imaging; LDL-C: low-density lipoprotein cholesterol; MDS-UPDRS: Movement Disorder Society-sponsored revision of the Unified Parkinson’s Disease Rating Scale; PICO: population, intervention, comparison, outcome; POINT: Platelet-Oriented Inhibition in New TIA and Minor Ischemic Stroke; TIA: transient ischemic attack.

Study	Population	Intervention/index approach	Comparator	Outcomes
Wang et al. [15]	Elderly patients with hip fracture undergoing general anesthesia	Perioperative intranasal insulin	Control/placebo or standard perioperative care	Incidence of postoperative delirium, perioperative cognitive outcomes, safety-related outcomes
Singh et al. [16]	Patients with acute ischemic stroke enrolled in the ESCAPE-NA1 trial	Assessment of infarcts in a new vascular territory after endovascular therapy	Patients without infarcts in a new territory or standard trial comparison groups	Frequency of new-territory infarcts, clinical outcomes, functional recovery, stroke-related prognosis
Piotrowski et al. [17]	Adult patients with treatment-naïve or recurrent/refractory glioblastoma	Pamiparib combined with radiation therapy and/or temozolomide	Standard treatment context, dose-escalation/dose-expansion comparison, or historical clinical expectations	Safety, tolerability, dose-limiting toxicity, progression-free survival, overall survival, treatment response
Guo et al. [18]	Patients with recurrent glioblastoma receiving immune checkpoint inhibition and low-dose bevacizumab	Tumor in situ fluid circulating tumor DNA analysis as a biomarker-monitoring and prognostic approach	Clinical outcome groups or biomarker-negative/lower-risk groups	Prognostic association, immunotherapy response, survival-related outcomes, and biomarker-monitoring performance
Xia et al. [19]	Patients with Alzheimer’s disease across multiple cohorts	Hippocampal radiomics model and associated biological analysis	Non-Alzheimer’s disease controls or comparative diagnostic/prognostic cohorts	Diagnostic/prognostic radiomics performance, hippocampal imaging signatures, biological pathway associations
Shokati et al. [20]	Patients with secondary progressive multiple sclerosis	Placenta-derived mesenchymal stem cell therapy	Baseline status or pre-treatment clinical/imaging/immunological measures	Safety, feasibility, EDSS changes, cognitive and psychological outcomes, DTI/fMRI findings, cytokine and B-cell marker changes
Kamel et al. [21]	Patients with high-risk transient ischemic attack or minor ischemic stroke in the POINT trial	Assessment of newly diagnosed atrial fibrillation after index TIA or minor ischemic stroke	TIA versus minor ischemic stroke groups	New atrial fibrillation diagnosis, cumulative AF risk, recurrent ischemic stroke, implications for rhythm monitoring
Pagano et al. [22]	Patients with early-stage Parkinson’s disease enrolled in the PASADENA open-label extension	Prasinezumab treatment, early-start or delayed-start	External comparator cohort from the Parkinson’s Progression Markers Initiative	Motor progression by MDS-UPDRS Part III OFF/ON states, MDS-UPDRS Part II, Hoehn and Yahr progression, and long-term disease progression
Gould et al. [23]	People living with motor neuron disease	Acceptance and commitment therapy plus usual care	Baseline status; no formal control group	Feasibility, acceptability, therapy engagement, quality of life, anxiety, depression, psychological flexibility, caregiver outcomes
Pozo-Rosich et al. [24]	Adults with episodic migraine	OnabotulinumtoxinA 155 U or 195 U	Placebo	Monthly migraine days, monthly headache days, 50% responder rate, acute medication use, patient-reported outcomes, adverse events
Liu et al. [25]	Patients with acute non-cardiogenic ischemic stroke with or without large artery atherosclerosis	Evolocumab plus atorvastatin	Atorvastatin monotherapy	Early neurological deterioration within 7 days, LDL-C target achievement, inflammatory markers, 90-day modified Rankin Scale outcome, recurrent stroke, adverse events
